# Placental extract ameliorates non-alcoholic steatohepatitis (NASH) by exerting protective effects on endothelial cells

**DOI:** 10.1016/j.heliyon.2017.e00416

**Published:** 2017-09-27

**Authors:** Akihiro Yamauchi, Akiko Kamiyoshi, Teruhide Koyama, Nobuyoshi Iinuma, Shumpei Yamaguchi, Hiroyuki Miyazaki, Eiichi Hirano, Taiichi Kaku, Takayuki Shindo

**Affiliations:** a Department of Cardiovascular Research, Shinshu University Graduate School of Medicine, Matsumoto, Japan; b Japan Bio Products Co., Ltd., Tokyo, Japan

**Keywords:** Health Sciences, Metabolism, Pathology, Pharmaceutical science

## Abstract

Non-alcoholic steatohepatitis (NASH) is a severe form of fatty liver disease that is defined by the presence of inflammation and fibrosis, ultimately leading to cirrhosis and hepatocellular carcinoma. Treatment with human placental extract (HPE) reportedly ameliorates the hepatic injury. We evaluated the effect of HPE treatment in a mouse model of NASH. In the methione- and choline-deficient (MCD) diet-induced liver injury model, fibrosis started from regions adjacent to the sinusoids. We administered the MCD diet with high-salt loading (8% NaCl in the drinking water) to mice deficient in the vasoprotective molecule RAMP2 for 5 weeks, with or without HPE. In both the HPE and control groups, fibrosis was seen in regions adjacent to the sinusoids, but the fibrosis was less pronounced in the HPE-treated mice. Levels of TNF-α and MMP9 expression were also significantly reduced in HPE-treated mice, and oxidative stress was suppressed in the perivascular region. In addition, HPE dose-dependently increased survival of cultured endothelial cells exposed to 100 μM H_2_O_2_, and it upregulated expression of eNOS and the anti-apoptotic factors bcl-2 and bcl-xL. From these observations, we conclude that HPE ameliorates NASH-associated pathologies by suppressing inflammation, oxidative stress and fibrosis. These beneficially effects of HPE are in part attributable to its protective effects on liver sinusoidal endothelial cells. HPE could thus be an attractive therapeutic candidate with which to suppress progression from simple fatty liver to NASH.

## 1 Introduction

Non-alcoholic fatty liver disease (NAFLD) is a common chronic liver disease and a major indicator of metabolic syndrome (Pacifico et al., 2011). Non-alcoholic steatohepatitis (NASH) is a more severe form of NAFLD that is broadly defined by the presence of steatosis with inflammation and progressive fibrosis, ultimately leading to cirrhosis and hepatocellular carcinoma (Fassio et al., 2004; Yatsuji et al., 2009). The numbers of NAFLD and NASH patients is increasing worldwide; consequently, understanding its pathology and developing a proper therapeutic approach are now important issues in clinical medicine. Although several therapeutic strategies have been attempted in efforts to treat NASH, there is as yet no consensus on how to evaluate NASH patients or treat them (Arab et al., 2014).

For more than 40 years, human placental extract (HPE) has been prescribed clinically to treat chronic hepatitis, liver cirrhosis and other hepatic diseases. In experimental animal model of hepatitis, HPE reportedly ameliorates hepatic injury through liver regeneration and inhibition of inflammatory reactions and hepatocyte apoptosis (Jung et al., 2011; Wu et al., 2008). Moreover, Shimokobe et al. recently reported that HPE is effective in NASH patients who were unresponsive to lifestyle intervention (Shimokobe et al., 2014). They treated patients with Laennec, a HPE formulation, for 8 weeks, and obtained significant reductions in serum transaminases (AST and ALT). Although body weight, lipid profiles and insulin resistance were unaffected by HPE, improvements in lobular inflammation and hepatocyte ballooning were noted in liver biopsies. The histological efficacy of HPE was better in obese patients than in non-obese ones, and no adverse events were observed during the study. Because it is safe and well tolerated, those investigators suggested that use of HPE was a potentially effective approach to the treatment of NASH. However, the mechanisms underlying the therapeutic effects of HPE were not explored.

Like other lifestyle-related diseases, multiple genetic and environmental components are associated with the development of NASH, though the mechanisms underlying its onset and progression are still unclear. According to the “two-hit hypothesis” for NASH, obesity is the “first hit” and the resultant overload of lipids consisting primarily of triglyceride (TG) and free fatty acid (FFA) induces hepatic steatosis (Day and James, 1998). There have been several suggestions as to the “second hit,” which leads to steatohepatitis and fibrosis (Ni et al., 2016). Among the various candidates involved in the second hit leading to NASH progression, we have focused on the liver sinusoidal endothelial cells (LSECs) (Arai et al., 2011; Iinuma et al., 2010). LSECs have unique structural and functional characteristics that differ from other vascular endothelial cells. LSECs have fenestrae, minimal basement membranes and loose cell-cell junctions, all of which contribute to efficient nutrient and gas exchange in the liver (Enomoto et al., 2004). LSECs also exhibit greater endocytotic activity than other types of endothelial cells (Smedsrod et al., 1990), and they express scavenger receptors such as the mannose receptor, Fc-receptor and stabilin-2, which may protect the parenchymal hepatocytes by scavenging toxic molecules. In association with liver fibrosis, however, LSECs undergo marked structural changes, with a reduction in fenestrae and development of basement membranes in a process called capillarization (Schaffner and Poper, 1963). It has been reported that hepatic injury begins with damage to the LSECs (Caldwell-Kenkel et al., 1989; McKeown et al., 1988), which suggests that protecting LSECs from injury could be a means to preserve liver function. In the present study, therefore, we evaluated the effectiveness of HPE in an animal model of NASH, focusing in particular on the effect of HPE treatment on LSECs.

## 2 Materials and methods

### 2.1 Animals

Mice were maintained under specific pathogen-free conditions in an environmentally controlled clean room at the Division of Laboratory Animal Research, Department of Life Science, Research Center for Human and Environmental Sciences, Shinshu University. All animal handling procedures were performed in accordance with protocols approved by the Ethics Committee of the Institutional Animal Care and Use Committee and NIH guidelines (Guide for the Care and Use of Laboratory Animals). Before all operative procedures, mice were anesthetized by intraperitoneal injection of 2,2,2-tribromoethanol (240 mg/kg; Wako, Osaka, Japan).

Wild-type C57BL/6J male mice were purchased from a supplier of experimental animals (CLEA Japan, Inc. Tokyo, Japan). RAMP2 knockout mice were originally generated by our group and are pure C57BL/6J background (Ichikawa-Shindo et al., 2008). Because homozygous RAMP2 knockout is embryonically lethal, we used 10 week-old male heterozygous RAMP2 knockout mice (RAMP2+/−) in this study.

### 2.2 NASH model

Methionine- and choline-deficient (MCD) diet (calorie ratios for carbohydrates, protein, and lipid were 58.2%, 18.3% and 23.5%) was purchased from Oriental Yeast Co., Ltd. (Tokyo, Japan). Mice were fed a normal diet until they were 10 weeks old. They were then challenged with the MCD diet and salt-loading (8% NaCl in drinking water) for 5 weeks, from ages 10 to 15 weeks. The HPE used in this study was hydrolysate of human placenta, or Laennec (Japan Bio Products Co., LTD, Tokyo, Japan). Mice were intramuscularly administered 0.1 ml of Laennec (3.6 mg/kg) or control saline twice a week during the challenge period. Under anesthesia, blood and tissue samples were collected at the end of the study. Serum transaminases, alanine aminotransferase (ALT) and aspartate aminotransferase (AST), were measured by a subcontractor (SRL, Tokyo, Japan).

CCl4 and concanavalin A-induced hepatitis models were generated as described previously (Kamiyoshi et al., 2006; Kamiyoshi et al., 2009).

### 2.3 Indocyanine green test

Indocyanine green (Daiichi-Sankyo, Tokyo, Japan) was intravenously injected at 1 mg/body. After 15 min, the mice were sacrificed and their livers were excised and fixed in 10% formalin.

### 2.4 Vascular casting of the liver

Under anesthesia, mice underwent laparotomy and about 500 μl of acrylic resin was injected through the portal vein. After 30 to 60 min, the injected acrylic resin had set. The liver was then excised, and the tissue was dissolved in 10% NaOH. The vascular cast was visualized using a digital microscope (Keyence, Osaka, Japan).

### 2.5 Histology and immunohistochemistry

Livers excised from mice were fixed for 24–48 h in 4% paraformaldehyde and embedded in paraffin, after which the tissues were cut into 5-μm-thick sections. The sections were used for silver staining or immunohistochemistry. For immunohistochemical analysis, the liver sections were incubated first with rabbit anti-rat p67-phox (MILLIPORE-UPSTATE, Billerica, MA) or mouse anti-mouse 4-hydroxy-2-nonenal (4HNE) (NOF Corporation, Tokyo, Japan), and then with Alexa Fluor 568 or 488-conjugated secondary antibody (ThermoFisher SCIENTIFIC, Waltham, MA). DAPI (Invitrogen, Carlsbad, CA) was used to stain the nuclei. Fluorescence was observed using a fluorescence microscope equipped with the appropriate filter sets (BZ-9000, KEYENCE, Osaka, Japan).

### 2.6 Cell viability assays

EAhy926 endothelial cells, an immortal, clonally pure, human endothelial cell line obtained through hybridization of HUVECs and line A 549/8 lung carcinoma cells, were kindly provided by C.J. Edgell, University of North Carolina, Chapel Hill, North Carolina, USA. EAhy926 human endothelial cells were cultured in DMEM (Invitrogen, Carlsbad, CA) supplemented with 10% FBS (Equitech-Bio Inc. Kerrville, TX) until sub-confluent, after which 100 μM H_2_O_2_ was added to the culture medium with or without Laennec. Then after incubation for an additional 24 h, the percent cell survival was calculated.

### 2.7 LSEC isolation and culture

Primary adult mouse LSECs were isolated using a two-step collagenase perfusion and centrifugation method described previously (Iinuma et al., 2010). LSECs were isolated from the supernatant after the first centrifugation. The isolated LSECs were then cultured in endothelial cell basal medium-2 (EBM-2) (Cambrex, Walkersville, MD).

### 2.8 Transmission electron microscopy

Specimens were fixed in 2.5% glutaraldehyde (pH 7.2), embedded in epoxy resin (Epok) 812 (Oken Shoji, Tokyo, Japan), cut into ultrathin sections, stained with uranyl acetate and lead citrate, and examined in an electron microscope.

### 2.9 Quantitative real-time RT-PCR analysis

Total RNA was isolated from tissues or cultured cells using a PureLink RNA Mini Kit (Thermo Fisher Scientific). RNA quality was verified using electrophoresis, and concentrations were measured using an Oubit 3.0 Fluorometer (Thermo Fisher Scientific). Thereafter, the extracted RNA was treated with DNA-Free (Thermo Fisher Scientific) to remove contaminating DNA, and 2-μg samples were subjected to reverse transcription using a PrimeScript™ RT reagent Kit (Takara Bio, Shiga, Japan). Quantitative real-time RT-PCR was carried out using a StepOne Plus Real-Time PCR System (Thermo Fisher Scientific) with SYBR green (Toyobo, Osaka, Japan) or Realtime PCR Master Mix (Toyobo) and TaqMan probe (MBL, Nagoya, Japan). Values were normalized to mouse and human GAPDH (Pre-Developed TaqMan assay reagents, Thermo Fisher Scientific). The primers used are listed in Table 1.

**Table 1:** Primers used for quantitative real-time PCR.

Mouse quantitative real-time PCR primers		
IL-1β	Forward	CTACAGGCTCCGAGATGAACAAC
	Reverse	TCCATTGAGGTGGAGAGCTTTC
TNF-α	Forward	ACGGCATGGATCTCAAAGAC
	Reverse	AGATAGCAAATCGGCTGACG
VCAM1	Forward	CCCTGAATACAAAACGATCGC
	Reverse	CAGCCCGTAGTGCTGCAAG
TGF-β	Forward	CCCGAAGCGGACTACTATGC
	Reverse	TAGATGGCGTTGTTGCGGT
COL1A1	Forward	ATGGATTCCCGTTCGAGTACG
	Reverse	TCAGCTGGATAGCGACATCG
COL4A2	Forward	CACAACATCAACGATCCACCC
	Reverse	GAACCCCATGATGCCTTCCT
	Probe	AGCAAGGGATACCCGGCGTAATCTCA
MMP2	Forward	GTGACACCACGTGACAAGCC
	Reverse	TGGGAGCTCAGGCCAGAAT
MMP9	Forward	CAGCTGGCAGAGGCATACTTG
	Reverse	GCTTCTCTCCCATCATCTGGG
p67 phox	Forward	CAGACCCAAAACCCCAGAAA
	Reverse	AAAGCCAAACAATACGCGGT
p22 phox	Forward	GGCCATTGCCAGTGTGATCT
	Reverse	GCTCAATGGGAGTCCACTGC

Human quantitative real-time PCR primers		
eNOS	Forward	AGATCTCCGCCTCGCTCAT
	Reverse	AGCCATACAGGATTGTCGCC
bax	Forward	AGAGGATGATTGCCGCCGT
	Reverse	CAACCACCCTGGTCTTGGATC
bcl-2	Forward	TGCACCTGACGCCCTTCAC
	Reverse	AGACAGCCAGGAGAAATCAAACAG
bcl-xL	Forward	GTAAACTGGGGTCGCATTGT
	Reverse	TGCTGCATTGTTCCCATAGA

### 2.10 Statistical analysis

Values are expressed as means ± SEM. Student’s t test was used to determine the significance of differences between two groups. One-way ANOVA followed by Fisher’s PLSD was used to determine significant differences among three groups. Values of *p* < 0.05 or *p* < 0.01 were considered significant.

## 3 Results

### 3.1 MCD treatment damages LSECs in chronic liver injury models

**Fig. 1:**
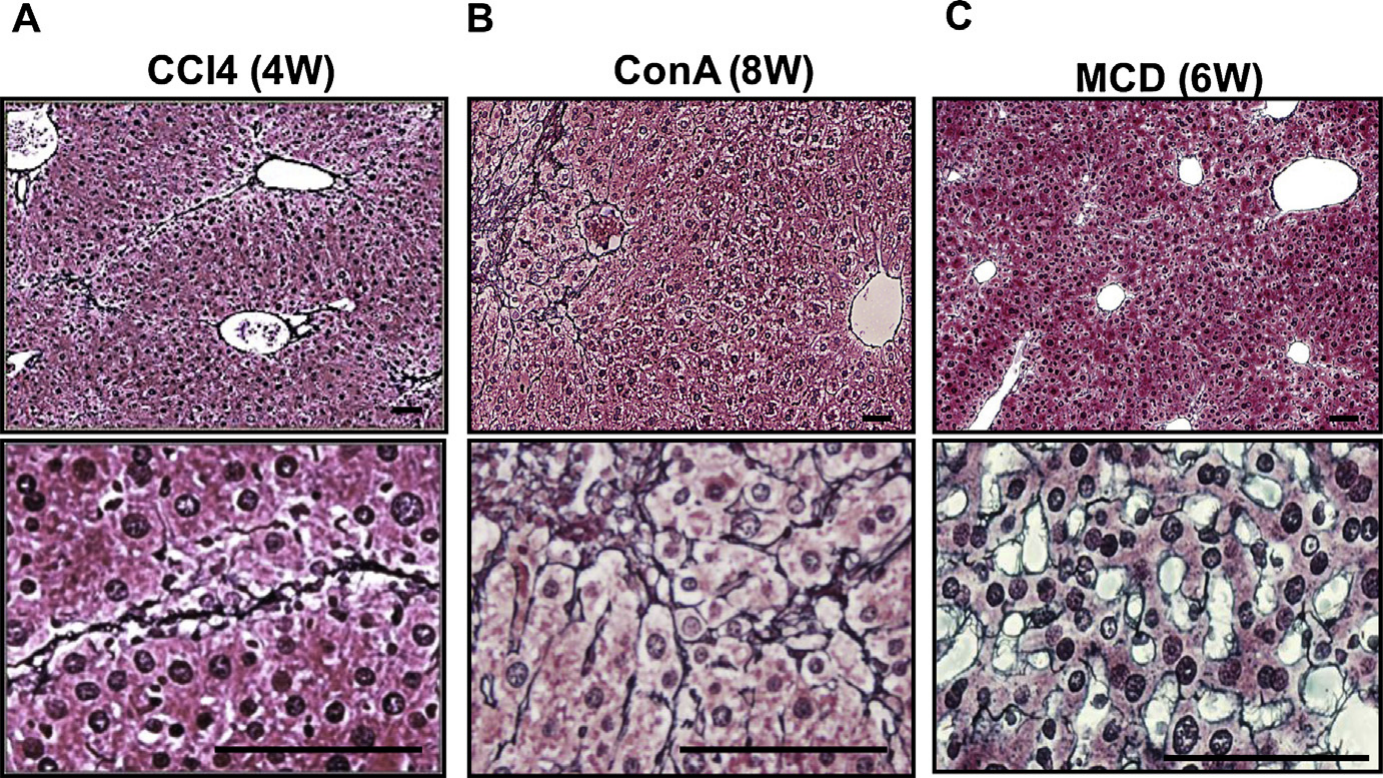
MCD diet caused fibrosis adjacent to the liver sinusoids. C57BL/6J wild-type mice were used for chronic liver injury models. Shown are silver-stained liver sections at lower (upper panels) and higher (lower panels) magnification. Silver-stained fibrotic areas were detected mainly between the parenchymal hepatocytes in CCl4-treated (A) and Con A-treated (B) mice, but were detected adjacent to the sinusoids in mice fed the MCD diet (C). Scale bars = 100 μm.

We initially generated several chronic liver injury models using wild-type mice and compared their pathological changes (Fig. 1A–C). Chronic administration of either carbon tetrachloride (CCl4) (4 weeks) (Fig. 1A) or concanavalin A (ConA) (8 weeks) (Fig. 1B) caused chronic liver injury with fibrosis, which was revealed by sliver staining of the liver sections. Fibrosis was also detected in the livers of mice fed the MCD diet (Fig. 1C), but the pattern of its progression differed from the other two models. After 6 weeks on the MCD diet, the overall fibrosis was less severe than in the other two models, but the fibrosis was mainly detected adjacent to the sinusoids. By contrast, the fibrosis was located between the parenchymal hepatocytes in mice treated with CCl4 or Con A.

**Fig. 2:**
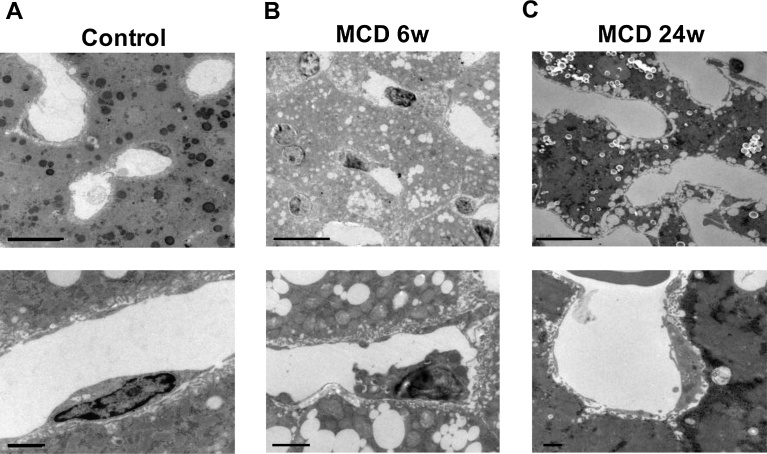
The MCD diet caused damage to LSECs. Electron microscopic observation of the liver sinusoids from control mice (A) and mice fed the MCD diet for 6 weeks (B) or 24 weeks (C). Upper and lower panels show lower and higher magnifications, respectively. The MCD diet caused fatty liver changes, characterized by the accumulation of lipid droplets within the hepatocytes (upper panels in B and C) as well as cellular deformities (lower panel in B) and nuclear loss (lower panel in C) in LSECs. Scale bars = 10 μm in upper panels, 1 μm in lower panels.

We speculated that the fibrosis starts from the vasculature in the MCD model, but arises to replace damaged and lost hepatocytes in the CCl4 and Con A models. This speculation was confirmed by electron microscopic observation of liver sinusoids (Fig. 2A–C). MCD treatment for 6 to 24 weeks caused fatty changes to the liver, which were characterized by the accumulation of lipid droplets within the hepatocytes (Fig. 2 upper panels). However, closer observation of the sinusoids revealed damage to LSECs (Fig. 2 lower panels). After 6 weeks of the MCD diet, we detected deformation of LSECs and vacuole formation; after 24 weeks, some LSECs had even lost their nuclei.

**Fig. 3:**
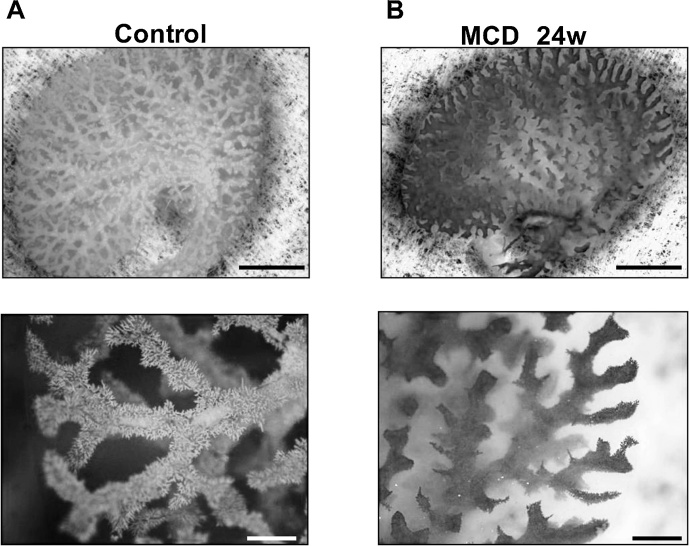
Vascular casting showed the MCD diet collapsed the structure of sinusoids. Acrylic resin casting of the liver vasculature from a control mouse (A) and a mouse fed the MCD diet for 24 weeks (B). Upper and lower panels show lower and higher magnifications, respectively. In control mice, sinusoidal casts were well visualized to the tip of each vascular branch (lower panel in A). In MCD-treated mice, the casting of the smaller vessels was indiscernible (lower panel in B). Scale bars = 5 mm in upper panels, 1 mm in lower panels.

**Fig. 4:**
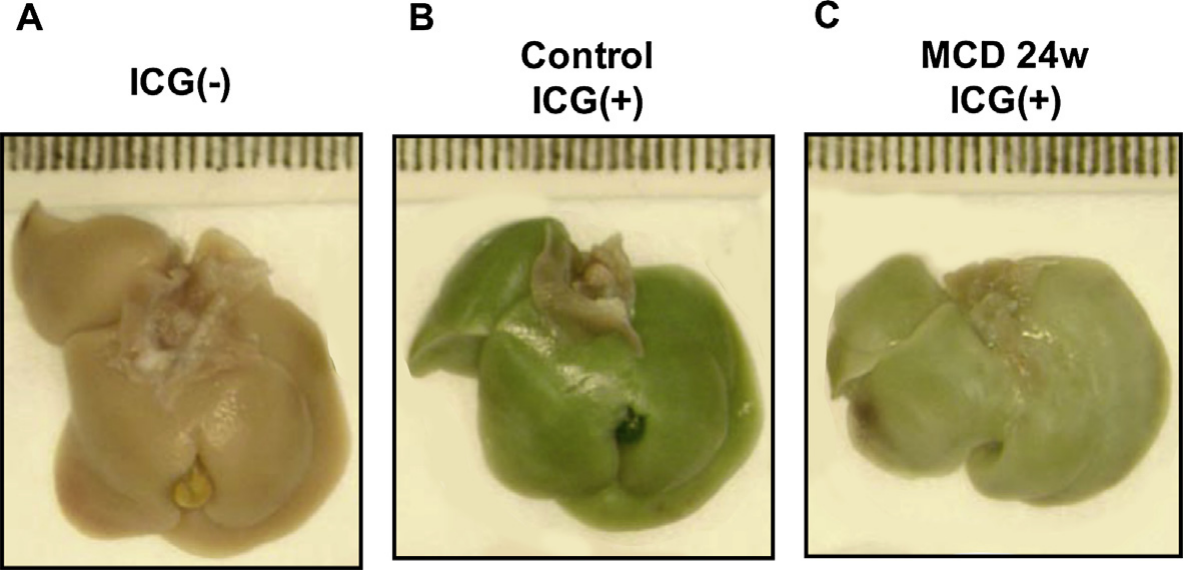
Indocyanine green (ICG) uptake was diminished in the livers of mice fed the MCD diet. Shown is the appearance of the liver without ICG injection (A) and after intravenous injection of ICG in control and (B) MCD-treated mice (C). Uptake of ICG was diminished in the MCD-treated mice (compare B and C).

The vascular abnormalities were also confirmed through vascular casting. In control mice, sinusoidal casting was readily detected to the tips of each vascular branch (Fig. 3, left panels). In the MCD-treated mice, acrylic resin injected from portal vein often did not reach the smaller peripheral vessels (Fig. 3, right panels). We also found that uptake of intravenously injected indocyanine green by parenchymal hepatocytes was diminished in MCD-treated mice (Fig. 4), which may be attributable to the impaired microcirculation within the liver. Based on these observations, we selected the MCD diet model to evaluate whether damage to the LSECs is the second hit in the NASH progression.

### 3.2 HPE treatment ameliorates liver injury

One of the drawbacks of the MCD diet model is that it takes a long time to induce chronic hepatitis in wild-type mice. In addition, long-term MCD treatment causes weight loss, which is clearly different from the situation in humans with fatty liver. We previously reported that RAMP2 is the key modulator of the endogenous vasoprotective peptide adrenomedullin (Ichikawa-Shindo et al., 2008), and that vascular endothelial cell-specific RAMP2 knockout mice show vascular inflammation and liver fibrosis (Koyama et al., 2013). Therefore, to accelerate the phenotypic changes observed with the MCD diet and shorten the treatment period, we used heterozygous RAMP2 knockout mice (RAMP2+/−) and administered MCD for 5 weeks along with 8% NaCl in the drinking water. During the MCD and high-salt loading, HPE or control saline was administered intramuscularly.

**Fig. 5:**
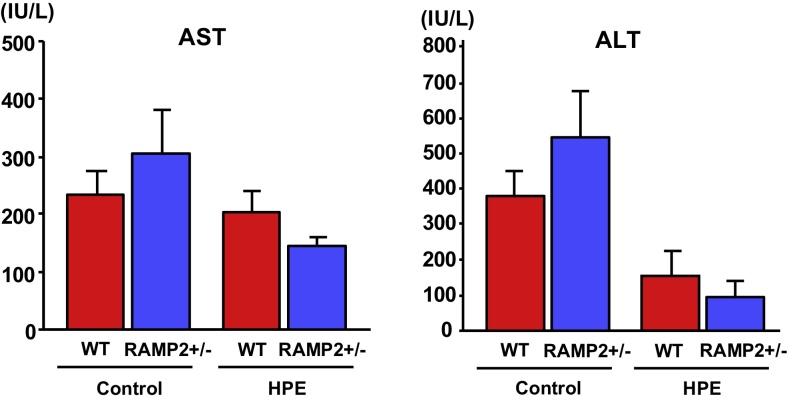
HPE treatment ameliorated liver injury. Serum AST and ALT levels in heterozygous RAMP2 knockout mice (RAMP2+/−) and wild-type mice administered the MCD diet for 5 weeks with 8% NaCl in the drinking water. Mice were treated with intramuscular injections of saline (Control) or HPE. Bars are means ± SEM. n = 8 in each group. Serum transaminases tended to be higher in the Control groups than in the HPE-treated groups.

**Fig. 6:**
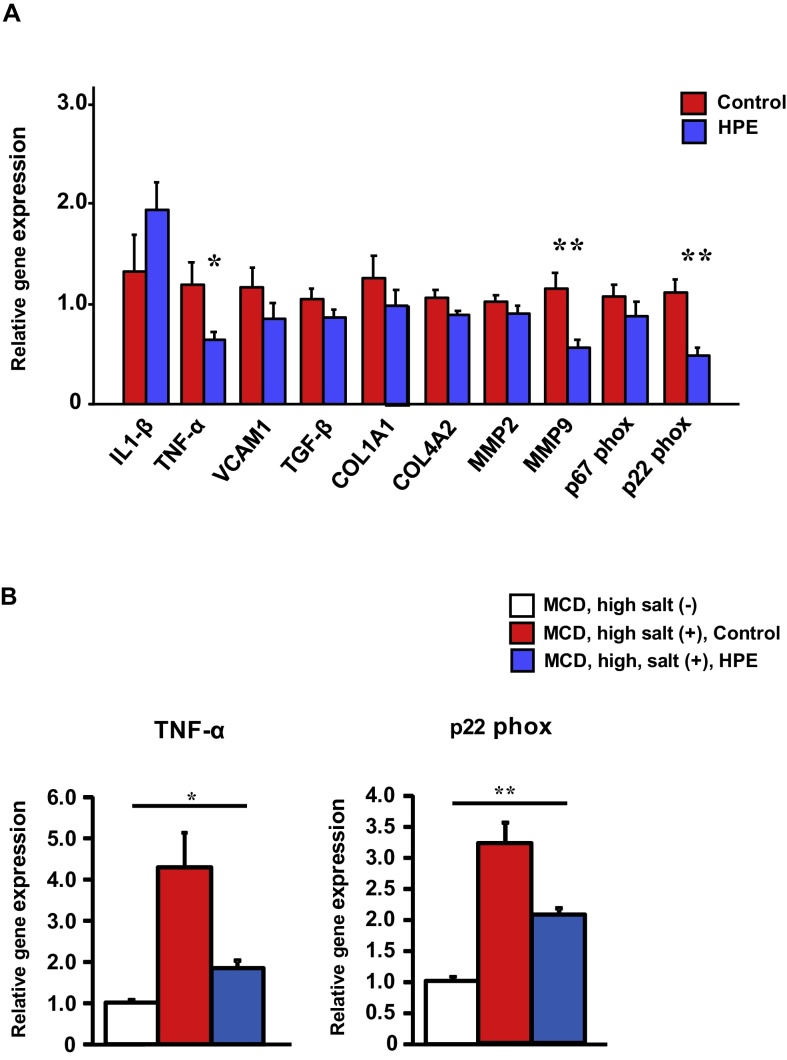
HPE treatment suppressed expression of inflammation-, fibrosis- and oxidative stress-related genes. A. Relative expression of the indicated genes in the livers of control and HPE-treated RAMP2+/− mice. The median of the control group was assigned a value of 1. Bars are means ± SEM. n = 8 in each group. *p < 0.05, **p < 0.01. HPE suppressed expression of inflammation-, fibrosis- and oxidative stress-related genes. B. Relative gene expression of TNF-α and p22phox in the livers of RAMP2+/− mice. The mean of the MCD, high salt (−) group was assigned a value of 1. Bars are means ± SEM. n = 8 in each group. *p < 0.05, **p < 0.01. Compared to the MCD, high salt (−) group, the MCD, high salt (+) group showed significantly elevated expression of both TNF-α and p22phox. HPE-treatment in MCD, high salt (+) group partially normalized expression of the inflammatory and oxidative stress markers.

Body weight, liver weight and the liver/body weight ratio were unchanged by HPE treatment. Serum transaminase levels (AST and ALT) were elevated to over 200 IU/L in the control groups of both wild-type and RAMP2+/− mice, but were reduced in HPE-treated mice (Fig. 5). Using real-time PCR analysis, we evaluated the expression of inflammation-, fibrosis- and oxidative stress-related genes (Fig. 6A). Expression of both TNF-α and MMP9 was significantly lower in HPE-treated mice than control mice, which may reflect less severe inflammation and tissue remodeling within the liver. Expression of p22 phox, a membrane-associated component of NADPH oxidase, was also significantly reduced in HPE-treated mice. In addition, when we compared gene expression between the MCD + high salt-treated and untreated groups (Fig. 6B), we found that TNF-α expression was 4-fold higher and p22phox expression was 3-fold higher in the MCD + high salt-treated than the untreated group. HPE treatment in MCD + high salt-treated group partially reversed the enhanced expression of these inflammatory and oxidative stress markers.

**Fig. 7:**
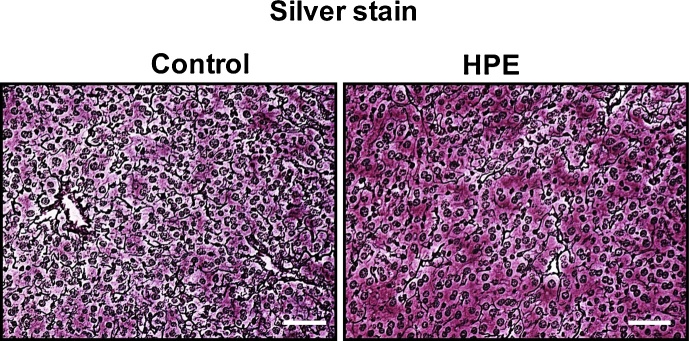
HPE treatment suppressed fibrotic changes in the livers of mice fed the MCD diet. Shown are sliver stained liver sections from representative control and HPE-treated mice. Silver stained fibrotic areas were detected adjacent to the sinusoids in both groups, but the areas were smaller in HPE-treated mice. Scale bars = 100 μm.

**Fig. 8:**
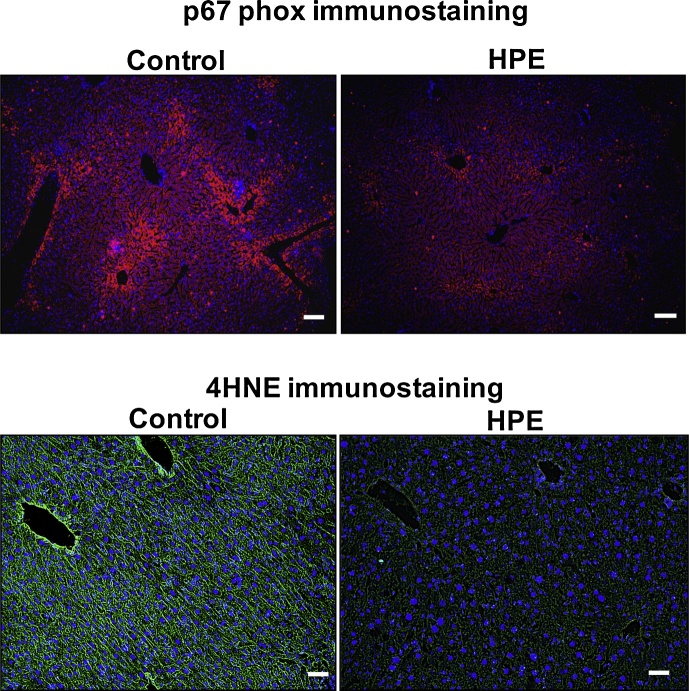
HPE-treatment suppressed oxidative stress in the livers of mice fed the MCD diet. Shown is immunostaining for p67 phox, a NADPH oxidase subunit (upper panels) and 4HNE, a lipid peroxidation product (lower panels), in liver sections. Immunostaining of p67 phox and 4HNE was detected in the perivascular regions of both control and HPE-treated mice, but it was much weaker in the HPE-treated group. Scale bars = 100 μm.

Silver staining revealed fibrotic areas adjacent to the sinusoids in both groups; however, the fibrosis was less severe in HPE-treated mice (Fig. 7). Using immunofluorescent staining, we observed strong expression of p67 phox, a cytosolic component of NADPH oxidase, in the perivascular regions of all mice, but the expression was less marked in HPE-treated mice (Fig. 8 upper panels). Levels of 4HNE, a lipid peroxidation product, were also decreased in HPE-treated mice (Fig. 8 lower panels). Taken together, these observations indicate that HPE treatment ameliorated liver injury by reducing inflammation, oxidative stress and fibrosis.

### 3.3 HPE treatment enhances endothelial cell survival by enhancing anti-apoptotic gene expression

**Fig. 9:**
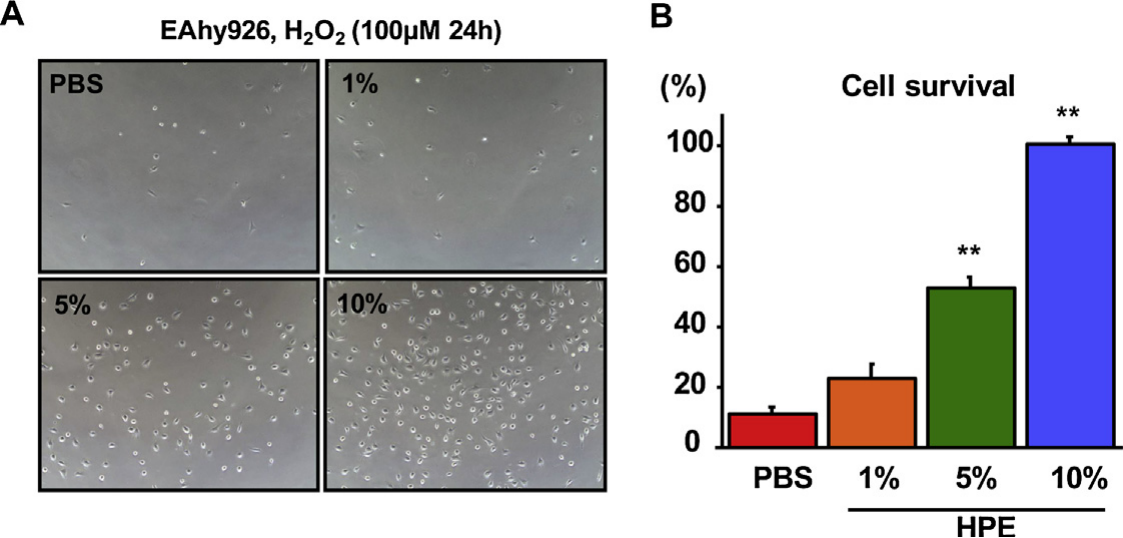
HPE treatment increased survival among cultured endothelial cells. EAhy926 human endothelial cells were exposed to 100 μM H_2_O_2_ for 24 h with or without HPE-treatment. Photos of the cultured endothelial cells (A) and the comparison of the cell survival (B) are shown. Bars are means ± SEM. The 10% HPE-treated group was assigned a value of 100%. n = 8 in each group. **p < 0.01. HPE dose-dependently increased endothelial cell survival.

**Fig. 10:**
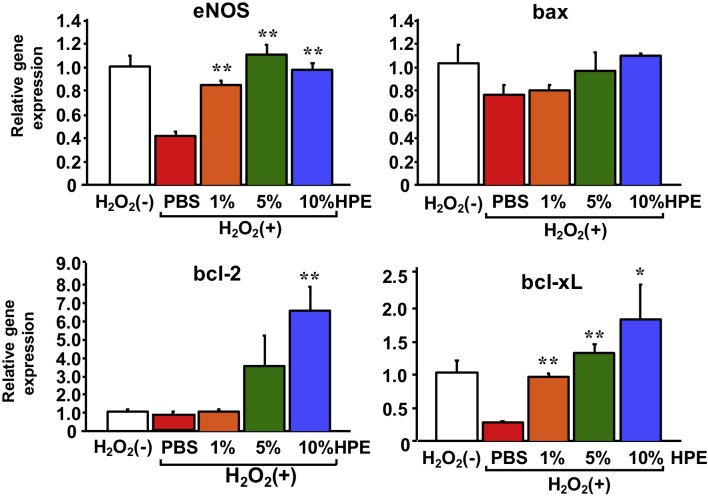
HPE-treatment upregulated expression of eNOS and anti-apoptotic genes. Expression of the indicated genes in cultured EAhy926 human endothelial cells. Bars are means ± SEM. The mean of the control H_2_O_2_(−) group was assigned a value of 1. n = 8 in each group. *p < 0.05, **p < 0.01. HPE-treatment upregulated expression of eNOS, reflecting increased endothelial cell viability. HPE-treatment also upregulated expression of the anti-apoptotic factors bcl-2 and bcl-xL, while expression of the pro-apoptotic factor bax was unchanged.

We next evaluated the direct effects of HPE on endothelial cells. HPE dose-dependently increased survival of EAhy926 endothelial cells exposed to 100 μM H_2_O_2_ (Fig. 9). Real-time PCR analysis showed that HPE upregulated eNOS expression, which may reflect the elevated endothelial cell viability. In an analysis of apoptosis-related factors, we found that the HPE treatment led to upregulated expression of two anti-apoptotic factors, bcl-2 and bcl-xL, while expression of the pro-apoptotic factor bax was unchanged (Fig. 10).

## 4 Discussion

There are currently no standard animal models that correctly reproduce the pathogenesis of NASH in humans, though several animal models have been proposed (Ibrahim et al., 2016). High-fat diet models are useful for mimicking the pathogenesis of obesity and the resultant metabolic disturbances. However, whereas long-term intake of a high-fat diet leads to obesity and fatty liver in mice, it does not evoke liver fibrosis, which is a defining histological feature of NASH. Therefore, one of our concerns in this study was to select the proper animal model with which to evaluate the effects of HPE on NASH.

CCl4-, ConA- and MCD diet-induced liver injury are all mouse models commonly used to evaluate the pathogenesis of chronic liver injury. Among them, we focused on the pattern of fibrosis within the liver. In the MCD diet model, fibrosis started from the region adjacent to the sinusoids, whereas in other two models it was mainly observed within the liver parenchyma. Therefore, to evaluate LSEC damage as the second hit of NASH progression, we selected the MCD model.

The MCD diet is high in sucrose and fat (40% sucrose, 10% fat), and is deficient in methionine and choline, which are essential for hepatic β oxidation and the production of very low-density lipoprotein (VLDL). In addition, the choline deficiency impairs hepatic VLDL secretion (Yao and Vance, 1990), increasing fat accumulation in the liver and decreasing VLDL synthesis. Subsequent oxidative stress and changes in cytokines in this model culminate in liver injury (Larter et al., 2008; Leclercq et al., 2000). We speculated that, as in human NASH, the MCD diet induces a two hit model in mice that results in a NASH-like condition: the first hit is lipid accumulation and the second hit the cellar injury caused by oxidative stress and inflammation.

Although the MCD diet model well replicates the histological features of the fibrosis observed in human NASH, its metabolic context differs from human NASH, as animals fed the MCD diet for prolonged periods lose body weight, show low blood glucose, low blood triglyceride (TG) and cholesterol, unchanged or increased serum adiponectin levels, and elevated peripheral insulin sensitivity, which is a metabolic profile opposite to the human disease (Larter et al., 2008). In the present study, therefore, we modified the MCD model by combining it with high-salt loading, because high salt intake would enhance liver damage and fibrosis in these mice (Wang et al., 2016). We also used RAMP2 knockout mice to shorten the feeding period by accelerating LSEC damage. Adrenomedullin is an endogenous vasoprotective peptide produced primarily by vascular endothelial cells (Kitamura et al., 1993), and RAMP2 is its receptor modulating protein (McLatchie et al., 1998). As we reported previously, the major vascular functions of adrenomedullin are regulated by RAMP2 (Ichikawa-Shindo et al., 2008). Thus, RAMP2 knockout mice develop spontaneous organ damage, which starts with vascular endothelial cell damage and vascular inflammation. Endothelial cell-specific RAMP2 knockout mice even show liver cirrhosis-like changes with aging (Koyama et al., 2013). Because homozygotic RAMP2 knockout is embryonically lethal (Ichikawa-Shindo et al., 2008), we used heterozygotes in this study. Five weeks of MCD and high-salt loading resulted in serum transaminase rising to over 200 IU/L and fibrotic changes occurring along the vasculature. These changes were suppressed by treatment with HPE. In addition, HPE suppressed expression of inflammation- and fibrosis-related genes and NADPH oxidase, especially in the perivascular regions. This suggests reductions in oxidative stress and inflammation are key beneficial effects of HPE.

In an earlier study, HPE was shown to suppress inflammation in a chronic arthritis rat model using complete Freund's adjuvant (Lee et al., 2011). Direct effects of HPE on the production of pro-inflammatory cytokines and mediators have also been reported. For example, HPE reportedly inhibits production of nitric oxide, TNF-α and cyclooxygenase-2 in lipopolysaccharide-stimulated RAW264.7 macrophages (Chen et al., 2012). In the present study, we found that HPE significantly suppressed TNF-α expression in the MCD diet model. This suggests HPE may suppress the progression of chronic inflammation initiated by lipid accumulation within hepatocytes.

In addition to inflammation, oxidative stress likely also contributes to chronic liver injury. In that regard, HPE showed both anti-oxidative and anti-inflammatory activities in rats exposed to benzo[a]pyrene (BaP) (Park et al., 2010). Application of H_2_O_2_ to cultured cells is used to evaluate the cellular damage caused by oxidative stress. In the present study, we found that HPE dose-dependently increased survival among endothelial cells exposed to H_2_O_2_. Accompanying the increased survival was upregulated expression of two anti-apoptotic factors, bcl-2 and bcl-xL, as well as eNOS, which may reflect anti-oxidative effects and elevated endothelial cell viability. LSEC injury appears during the simple steatosis phase, and precedes the appearance of activated Kupffer cells and hepatic stellate cells (Miyao et al., 2015). Thus LSEC injury may have a “gatekeeper” role in the progression of NASH.

There have been several hypotheses regarding the NASH second hit, which leads to steatohepatitis and fibrosis (Ni et al., 2016). Ingested free fatty acids and free cholesterol may induce endoplasmic reticulum stress and oxidative stress, leading to hepatic inflammation and fibrogenesis. Adipokines, such as IL-6 and TNF-α, produced during chronic inflammation of hypertrophied white adipose tissue are thought to cause hepatocyte fat accumulation and liver inflammation. Recently, an association between metabolic disorder and intestinal flora has attracted attention. Bacterial overgrowth occurring in the small intestine of NASH patients may result in the presence of lipopolysaccharide in the portal circulation and activation of Kupffer cells in the liver, which may in turn contribute the progression liver damage. In addition to these candidates, we propose that LSEC damage is another important potential second hit of the progression of NASH, and that LSEC protection could preserve liver function. At present, little is known about the mediators that directly modulate LSECs function. However, our findings suggest HPE may be an effective therapeutic candidate with which to suppress the second hit during the progression from simple NAFLD to NASH.

## 5 Conclusion

In summary, we showed that HPE ameliorates the pathology of MCD-induced NASH in mice by suppressing inflammation, oxidative stress and fibrosis. Furthermore, we found that HPE directly suppresses endothelial cell damage. HPE could thus be an effective therapeutic agent with which to suppress progression from simple fatty liver to NASH.

## Declarations

### Author contribution statement

Akihiro Yamauchi: Performed the experiments; Analyzed and interpreted the data; Contributed reagents, materials, analysis tools or data.

Akiko Kamiyoshi, Teruhide Koyama, Nobuyoshi Iinuma: Performed the experiments; Analyzed and interpreted the data.

Shumpei Yamaguchi, Hiroyuki Miyazaki, Eiichi Hirano, Taiichi Kaku: Conceived and designed the experiments.

Takayuki Shindo: Conceived and designed the experiments; Wrote the paper.

### Competing interest statement

The authors declare no conflict of interest.

### Funding statement

This work was supported by Japan Bio Products Co., Ltd., as a collaborative project.

### Additional information

No additional information is available for this paper.
